# Residential Consumer-Centric Demand-Side Management Based on Energy Disaggregation-Piloting Constrained Swarm Intelligence: Towards Edge Computing

**DOI:** 10.3390/s18051365

**Published:** 2018-04-27

**Authors:** Yu-Hsiu Lin, Yu-Chen Hu

**Affiliations:** Department of Computer Science and Information Management, Providence University, No. 200, Sec. 7, Taiwan Boulevard, Shalu Dist., Taichung City 43301, Taiwan; ychu@pu.edu.tw

**Keywords:** demand-side management, demand response, edge computing, energy disaggregation, swarm intelligence

## Abstract

The emergence of smart Internet of Things (IoT) devices has highly favored the realization of smart homes in a down-stream sector of a smart grid. The underlying objective of Demand Response (DR) schemes is to actively engage customers to modify their energy consumption on domestic appliances in response to pricing signals. Domestic appliance scheduling is widely accepted as an effective mechanism to manage domestic energy consumption intelligently. Besides, to residential customers for DR implementation, maintaining a balance between energy consumption cost and users’ comfort satisfaction is a challenge. Hence, in this paper, a constrained Particle Swarm Optimization (PSO)-based residential consumer-centric load-scheduling method is proposed. The method can be further featured with edge computing. In contrast with cloud computing, edge computing—a method of optimizing cloud computing technologies by driving computing capabilities at the IoT edge of the Internet as one of the emerging trends in engineering technology—addresses bandwidth-intensive contents and latency-sensitive applications required among sensors and central data centers through data analytics at or near the source of data. A non-intrusive load-monitoring technique proposed previously is utilized to automatic determination of physical characteristics of power-intensive home appliances from users’ life patterns. The swarm intelligence, constrained PSO, is used to minimize the energy consumption cost while considering users’ comfort satisfaction for DR implementation. The residential consumer-centric load-scheduling method proposed in this paper is evaluated under real-time pricing with inclining block rates and is demonstrated in a case study. The experimentation reported in this paper shows the proposed residential consumer-centric load-scheduling method can re-shape loads by home appliances in response to DR signals. Moreover, a phenomenal reduction in peak power consumption is achieved by 13.97%.

## 1. Introduction

A smart city in brief can be defined as a city in which Information and Communication Technologies (ICT) such as smart sensing, cognitive learning as well as context-aware computing are employed to make lives more comfortable and sustainable [1]. The Internet of Things (IoT), being designed and used to respond to needs for real-time and context-specific information intelligence and analytics to address specific local imperatives [2], is a key enabler for smart cities. Management of smart and green buildings and houses in downstream sectors of a smart grid often requires analyzing IoT data from inter-connected end-sensing devices and actuators, to optimize electrical efficiency and comfort satisfaction. Smart grid techniques, which conduct ICT to upgrade a traditional power grid into a smart one, are being developed to gains of energy inefficiencies by consumers where residential households have a significant role in energy consumption and threaten the power grid in grid health and power reliability. From smart and green buildings and houses in downstream sectors of a smart grid, significant efficiency gains make cities sustainable in terms of resources. The realization of smart, energy-efficient as well as green home infrastructure will form the backbone of future green city architecture [3]. Therefore, energy management covering Demand-Side Management (DSM), peak load reduction, and carbon emissions reduction [4] in smart buildings and houses is a key aspect of building efficient smart cities [5]. As pointed out in [6], there has been evidence from various load surveys that the demand of electricity in residential and commercial buildings is highly variable and changes throughout the day. Hence, conducting efficient energy management of home demands as well as reducing peak energy demands meet the rapidly growing need of building efficient smart cities. In contrast with increasing the maximum power generation capacity by more required power generators of power plants in a smart grid, reducing peak loads is mostly valuable for utilities to meet the increased energy demands. Demand Response (DR) strategies that motivate consumers to re-shape load profiles as well as limit peak energy demands by home appliances through smart meters based on time-based rates are gaining an importance in a smart grid, due to continuously increasing energy demands by consumers. Both the consumers that respond to DR signals in exchange for a discount on electricity prices and the utilities that ensures the un-jeopardized stability of the smart grid can benefit from DR as DSM.

In the literature, several advanced technical optimization approaches that deal with residential DR for smart homes in a smart grid have been developed, where (1) heuristic-based load control strategies for diverting peak power consumption [6] and shedding household appliances [7] and (2) load-scheduling methods for scheduling power consumption on household appliances [8,9,10,11,12,13,14,15,16,17,18] have been proposed. The authors have attempted to address residential DR. Nevertheless, most of the approaches do not consider Real-Time Pricing (RTP) with Inclining Block Rates (IBR). Compared with the Time-Of-Use (TOU) model where the variant of dynamic pricing establishes a variable electricity prices structure for peak, shoulder, and off-peak hours, RTP has been identified as the popular variant of dynamic pricing for DR implementation in a future grid as indicated in [18]. IBR can diminish peak energy demands. Besides, before taking the advantage of RTP in DR, consumers need to first determine the physical characteristics of household appliances based on their past trends of using electricity manually. They do not pay attention to automated residential DR; user intervention to the approaches is required. Also, all the approaches in the literature do not consider locally generated renewable energy such as photovoltaic power generation and/or wind power generation during residential DR, as renewable energy has the advantage that energy is clean and abundantly available in nature. In [6], the study mainly focuses on proposing a home energy management system, Home Energy Management as a Service, constituting reinforcement learning with four peak reduction thresholds and being interactive with the smart environment. In [9], a heuristic-based load-scheduling method that optimizes the electricity cost was realized. However, in the research, the peak power consumption leading to a relatively high Peak-to-Average Ratio (PAR) may emerge when the considered electricity price is low. Thus, IBR should be considered and included for residential DR implementation. In [10], both the electricity cost and PAR can be simultaneously reduced. However, the assumptions made in the study seem impractical, as indicated in [8]. To alleviate the defects in [9], reference [8] proposes a Genetic Algorithm (GA)-based load-scheduling approach optimizing household appliances based on RTP with IBR. In contrast with TOU establishing an electricity price-varying model for peak, shoulder, and off-peak hours, RTP is identified and expected as the popular variant of dynamic pricing for DR implementation in smart grids. With proposed in [8], residents who conduct and use the load-scheduling approach to optimize their electricity cost need to manually specify the physical characteristics of each enrolled household appliance in advance. In [10,11,12], the authors considered the energy consumption cost as the primary objective function of DSM. Besides, in the research, the IBR that diminishes the peak power consumption is not taken into consideration. In [13], the authors developed a recursive process on four load control scenarios for reduction of peak power computation. During the recursive process in [13], RTP is considered. In [14], a variant of ant colony optimization is used to solve the DSM problem. The research in [15] against most up-to-date studies in non-intrusive load monitoring [16,17] as a part of DSM takes into consideration RTP with IBR for DR implementation. However, the PAR reported in the research could be improved. Assuming an advanced building energy management system for air-conditioning facilities in commercial buildings, the researchers in [18] study a simulated annealing optimization that minimized an evaluation function consisting of power cost and comfort degradation terms.

As motivated before and surveyed above, energy efficiency is one of the central issues in smart and green buildings and houses. Intelligent energy management encompassing modern IoT technologies and data science analytics to re-shape load profiles as well as reduce peak energy demands by home appliances through smart meters based on time-based rates (a.k.a. dynamic pricing) unlocks the full potential of smart and green buildings and houses. In summary, the main objective of the work in this paper is to shift load profiles by home appliances as well as cut down on peak energy demands through a new constrained swarm intelligence-based residential consumer-centric DSM method considering predictable day-ahead RTP with IBR and comfort satisfaction of using electricity to consumers based on their past trends of gathered load data in a smart home environment with minimal user intervention. The new method modelled for residential DR, presented in this paper, and used to achieve the objective is (1) facilitated by energy disaggregation [15] for fully automated physical characteristics of household appliances, (2) mathematically formulated in a weighted-sum manner, and (3) executed for load scheduling under IBR-combined predictable day-ahead RTP signals. In this paper, locally generated renewable energy such as photovoltaic power generation and/or wind power generation is considered and mathematically formulated. This is because renewable energy has an advantage in which energy is clean as well as infinite in nature. RTP allowing electricity prices to change on an hourly time basis based on market demands is considered in this paper, since RTP identified and combined with IBR will be used as the popular dynamic pricing of DR implementation for future smart grids. As validated in this paper, a phenomenal peak energy demand reduction of 13.97% is achieved by the new constrained swarm intelligence-based residential consumer-centric DSM method. Major abbreviations/acronyms in this paper are defined in Table 1.

This paper is organized as follows. The residential consumer-centric DSM model used to realize DR programs to utilities is given in Section 2. Section 3 presents the proposed method. Experimentation is conducted in Section 4. Finally, Section 5 concludes this paper.

## 2. Residential Consumer-Centric Energy Management Model Involving Future Edge Computing

**Figure 1:**
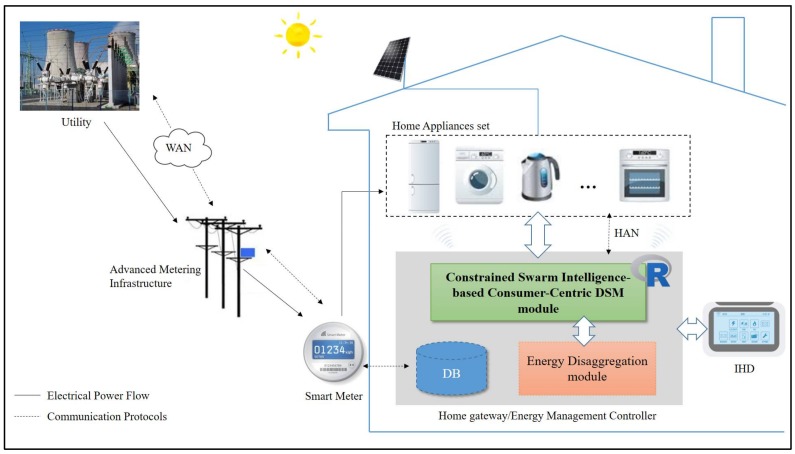
Schematic Diagram of the residential consumer-centric DSM model enabling utilities and consumers to operate their energy management schemes.

An Overview of the residential consumer-centric DSM model is depicted in Figure 1. The model can be further featured with edge computing where a method of optimizing cloud computing technologies by driving computing capabilities at the edge/IoT data sources of the Internet emerges as one of the emerging trends in engineering technology.

The model mainly comprises a smart meter used to receive DR signals from utilities via a wide area network/advanced metering infrastructure for DSM, a home gateway implemented on an embedded system in [15] or a laptop computer in this paper and acted as a well-known Energy Management Controller (EMC), and home appliances monitored and optimized for substantial electricity cost savings with consideration of user comfort preferences. A wireless communication network/wireless home area network constructed and used to forward gathered load information from monitored home appliances to the central EMC for further DR implementation. In contrast with cloud computing, edge computing, a method of optimizing cloud computing technologies by driving computing capabilities at the IoT edge of the Internet as one of the emerging trends in engineering technology, addresses bandwidth-intensive contents and latency-sensitive applications required among sensors and central data centers through data analytics at or near the source of data. To the home gateway in the proposed residential consumer-centric DSM model a BeagleBoard embedded system having an OMAP3530 720 MHz ARM^®^ Cortex^TM^-A8 processor (BeagleBoard, Texas Instruments, Dallas, TX, USA) can be featured, where an Apache HTTP server, MySQL Relational Database, PHP sous Linux OS stack can also be configured for the implementation and realization of edge computing as one of the emerging trends in engineering technology. The upgraded one can be applied to different IoT environments/use cases where (i) network connectivity is not always available or is limited and (ii) data need to be processed for real-time actions at the IoT data source. In this paper, the energy disaggregation developed in [15] is conducted for automated determination of operational characteristics of monitored home appliances from emerged human life patterns/load profiles. In this paper, the proposed constrained residential consumer-centric DSM method for electricity cost versus discomfort minimization along with peak demand reduction is implemented in R language. R language is a free software environment for statistical computing and graphics; it publicly provides a free package repository to feature more than 11,800 available software packages ranging from Machine Learning & Statistical Learning to Graphics for Data Science/Big Data analytics and data visualization [19]. In this paper, R language is also suited and used as a TCP/IP server (i) allowing engineering programs to use facilities of R from various programming languages without the need of initializing R or linking against R libraries and (ii) being able to start multiple R serves to handle multiple connections via different TCP/IP ports for concurrent R sessions [19]. For pairing R/SparkR with the EMC to Big Data analytics, we will demonstrate a more pragmatic approach against the method proposed in this paper in a Big Data analytics way.

## 3. Energy Disaggregation-Piloting Constrained Swarm Intelligence

To take advantage of DR programs such as minimization of electricity costs considering user satisfaction, residents need a home wizard to help them determine when their home appliances reacting to DR pricing must be scheduled based on their past trends.

In this section, the constrained swarm intelligence-based consumer-centric DSM method proposed in this paper is introduced. The proposed constrained swarm intelligence-based consumer-centric DSM method involves the following two stages. In the first stage, an energy disaggregation algorithm proposed in [15] is conducted and used to automatically characterize home appliances and consequently remove user intervention (there is no need to manually specify load characteristics/constraints to the objective function for DR implementation) based on historical data with past trends. In the second stage, a constrained swarm intelligence, constrained Particle Swarm Optimization (PSO), is executed for optimal schedules of monitored and enrolled home appliances in response to DR pricing once the home appliances are characterized through energy disaggregation.

### 3.1. Particle Swarm Optimization

In a smart grid, down-stream sectors have a huge number of different types of home appliances. The home appliances also have different load characteristics such as power ratings as natural signatures and operational constraints with various user comfort preferences. In literature, mathematical techniques such as linear programming can be employed and used to efficiently handle such the complexities. However, more computational resources are required, and they are inadequate to handle multiple constraints [20,21,22]. Meta-heuristics such as swarm intelligence, PSO, have been shown superior capabilities to cope with such the complexities. In this paper, the PSO used during the optimization process of the proposed residential customer-centric DSM model in this paper is briefly discussed below.

The conventional PSO [23,24,25,26,27] inspired by the swarming or collaborative behavior of biological populations and developed by Dr. Eberhart and Dr. Kennedy in 1995 is a population-based stochastic optimization technique. The PSO is similar to GA in the sense that these two meta-heuristics are population-based search methods. Compared with the GA solving engineering optimization problems that the search space is highly modal, discontinuous, and/or constrained [24], the PSO where the engineering optimization problems addressed by the GA can also be solved has the following three advantages: First, there are no explicit evolution operators—selection operations, crossover operations, and mutation operations. Second, it has low probabilities that solutions fall in local optimization regions. Third, the designed optimization process has fewer adjusted parameters than that of the GA. The PSO has been successfully applied in many engineering fields such as continuous function optimization and fuzzy logic control. The conventional PSO is initialized with a population of randomly generated particles as solution candidates. It searches for global optima by updating particles through iterations; particles having their own velocity to direct the flying of themselves fly through the search space by following the current optimal particles (it is the best strategy to find the best solution). In each generation of the optimization process, each particle is updated by *p_best_* and *g_best_*. Where, *p_best_* is the best solution (from the local view), which has achieved so far; *g_best_* is the best solution (from the global view) that the solution is obtained currently by any particle in the population. After finding *p_best_* and *g_best_*, the particle updates its velocity and position as follows.

Use (1) to update *particle velocity* “*v*[*t* + 1].”

*v*[*t* + 1] = *w* ∙ *v*[*t*] + *c*_1_ ∙ *rand*() ∙ (*p_best_*[*t*] − *present*[*t*]) + *c*_2_ ∙ *rand*() ∙ (*g_best_*[*t*] − *present*[*t*]). (1)

In Equation (1), *t* denotes the *t*-th iteration; *w* is the inertia weight parameter; *rand*() being a randomly generated real number belongs to (0, 1); *c*_1_ and*c*_2_ are acceleration factors. In this paper, *w* in Equation (1) varies as Equation (2).

*w* = *w_max_* − (*w_max_* − *w_min_*) ∙ (*Iteration_t_*/*Iteration_tmax_*). (2)

In Equation (2), *Iteration_tmax_* stands for the maximum number of iterations. Moreover, in this paper, coefficients *c*_1_ and *c*_2_ in Equation (1) are adaptively changed, which impacts on the convergence speed and optimization accuracy [19]; *c*_1_ and *c*_2_ are adapted by Equations (3) and (4) respectively during the optimization process.

*c*_1_ = *c*_1*max*_ − (*c*_1*max*_ − *c*_1*min*_) ∙ (*Iteration_t_*/*Iteration_tmax_*). (3)

*c*_2_ = *c*_2*max*_ − (*c*_2*max*_ − *c*_2*min*_) ∙ (*Iteration_t_*/*Iteration_tmax_*). (4)

Use Equation (5) to update *particle position* “*present*[*t* + 1].”

*present*[*t* + 1] = *present*[*t*] + *v*[*t* + 1]. (5)

From generation to generation, particles converge to the best solution. The pseudo code of the PSO procedure used to realize residential consumer-centric DSM in this paper is given in Algorithm 1. More variants such as swarm activity defined in [28] and used as the root mean square velocity of particles in PSO can also be conducted for examinations.
**Algorithm 1:** The PSO procedure with its variants proposed in [26]**For** each particle  *Randomly Initialize* the particle **End****Do**  For each particle   *Compute* its fitness value (the objective function optimized for residential consumer-centric DSM in this paper is described in Section 3.2)     If the fitness value is better than *p_best_* in history, then    *Set* the current value as the new *p_best_*
  End
 *Choose* the particle with the best fitness value (against all the other particles in the population) as the *g_best_*
  For each particle   *Compute* its particle velocity according to Equation (1)   *Update* its particle position according to Equation (5)   End
 During the optimization process, operational constraints by the objective function in Section 3.2 need to be satisfied. **While** the pre-specified maximum iteration or the minimum error tolerance is not attained  The goal of the constrained PSO used in this paper is to minimize electricity costs and maximize user satisfaction; at the same time, all the constraints are respected.

The residential consumer-centric DSM addressed in this paper and optimized by the PSO above is mathematically modeled in Section 3.2.

### 3.2. Load-Scheduling Formulation

The objective of the proposed residential consumer-centric DSM solved by the PSO in this paper is to simultaneously optimize electricity costs and user satisfaction while respecting all operational constraints of appliance models. An appliance model is described below.

Consider there are *n* schedulable home appliances monitored and enrolled for participation in DR programs. 1 day is divided into 24 h with a total of *T* time slots. A schedulable home appliance model can be represented by a tuple: (*α_i_*, *β_i_*, *l_i_*, *s_i_*, *δ_i_*). Where, [*α_i_*, *β_i_*] stands for the time interval in which the *i*-th schedulable home appliance in a smart home environment was identified and expected statistically for use through an analysis of energy disaggregation [15]. *l_i_* is the time duration of the *i*-th schedulable home appliance in which the load is present. Notice that *β_i_* − *α_i_* must be greater than or equal to *l_i_*. It must also be less than or equal to *T*. *s_i_* ranging from the time interval [*α_i_*, *β_i_* − *l_i_*] accounts for the time in which the *i*-th schedulable home appliance is started for use. By introducing a marginal parameter, *δ_i_*, *s_i_* of the *i*-th home appliance that is valid to be optimized falls into the time interval [*α_i_* − *δ_i_*, *β_i_* − *l_i_* + *δ_i_*].

Now, the mathematical formulation of the objective function that is subject to the operational constraints to the constrained swarm intelligence-based residential consumer-centric DSM in this paper is clarified as Equation (6).

(6) $$\begin{array}{ll} Minimize & w_{1}\cdot \sum_{h=1}^{H=24}[(\sum_{i=1}^{n}P_{rating}^{i}\cdot x_{h}^{i})-(P_{renewable}^{h}\cdot \Delta_{h})]\cdot \eta_{h}+w_{2}\cdot \sum_{i=1}^{n}\left|s_{i}-\alpha_{i}\right|\\ s.t. & s_{i}\in \left[\alpha_{i}-\delta_{i},\beta_{i}-l_{i}+\delta_{i}\right] \end{array}$$

Equation (6) shows the objective function to be optimized and composed of the electricity cost and the user satisfaction. In Equation (6), the two objectives are fused into one single objective in a weighted-sum way. The PSO simultaneously optimizes the two objectives. The two objectives can be assigned equal weights *w*_1_ and *w*_2_, respectively. The weight coefficients can vary from 0 to 1, and *w*_1_ + *w*_2_ = 1. *η_h_* in Equation (6) denotes the hourly electricity expense; it combines day-ahead RTP with five-level IBR. The five-level IBR used imposes more expense and diminishes PAR. It can be expected that IBR avoiding the outcome of peak demands during the optimization process restrains PAR so that the un-jeopardized stability of the power grid remains to utilities. In Equation (6), *P^i^_rating_* represents the power rating of the *i*-th home appliances. *x_h_^i^* identifying whether the *i*-th home appliance is being operated for use in period *h* or not belongs to {0, 1}. The constraints in Equation (6) imply that operational length of scheduled home appliances in time duration is all complete/satisfactory to avoid the users’ frustration. That is, the home appliances scheduled fulfill their operation time. In the first objective of Equation (6), must-run service of home appliances is considered. Also, the first objective is dually altered by a term, *P^h^_renewable_*∙∆*_h_*, of locally generated renewable energy resources, where local renewable energy such as photovoltaics with output power of *P^h^_renewable_* is generated and identified in time period of ∆*_h_* for load scheduling. In Equation (6), the second objective considering user satisfaction of using electricity is a shifted control of |*s_i_* − *α_i_*|. *δ_i_* accounts for the marginal parameter that the *i*-th schedulable home appliance falling into the time interval [*α_i_* − *δ_i_*, *β_i_* − *l_i_* + *δ_i_*] is valid to be scheduled/optimized.

Through the constrained optimization process, it is depicted that the total electricity cost must be less than that of the original one. Moreover, with or without use of the proposed constrained PSO-based residential consumer-centric DSM method, the total energy consumption on the home appliances must be the same.

In this paper, the following two constraints are also made and satisfied during the constrained optimization process. First, the scheduled home appliances are not interruptible. Second, during the optimization process, the total ampacity must not exceed the ampere capacity of the circuit breaker(s) in the main electrical panel of the household.

## 4. Case Study

**Figure 2:**
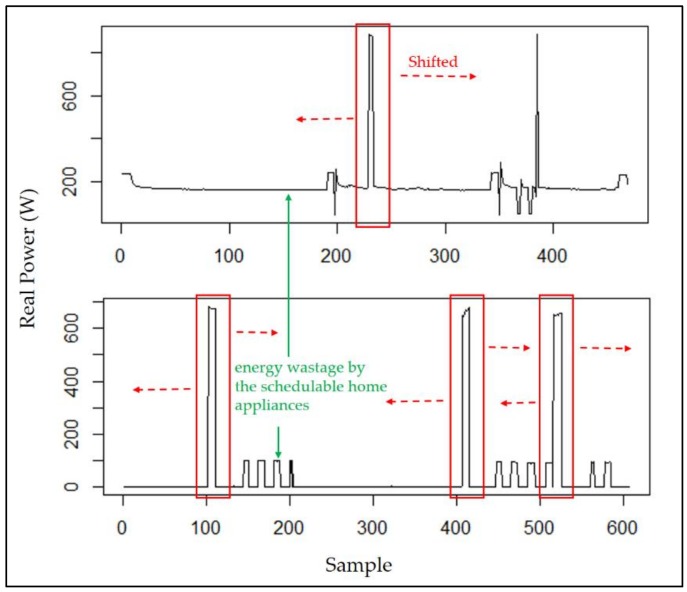
Load profile of electric water boilers.

**Figure 3:**
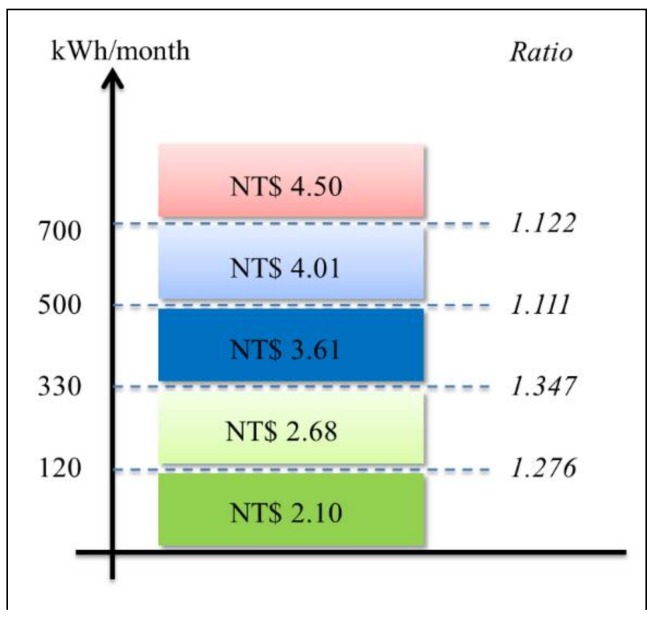
Five-level IBR used in this paper and announced by Taipower , a state-owned electric power industry (Taiwan Power Company, Taipei City, Taiwan) providing electricity to Taiwan and offshore islands of the Republic of China, in Taiwan.

**Figure 4:**
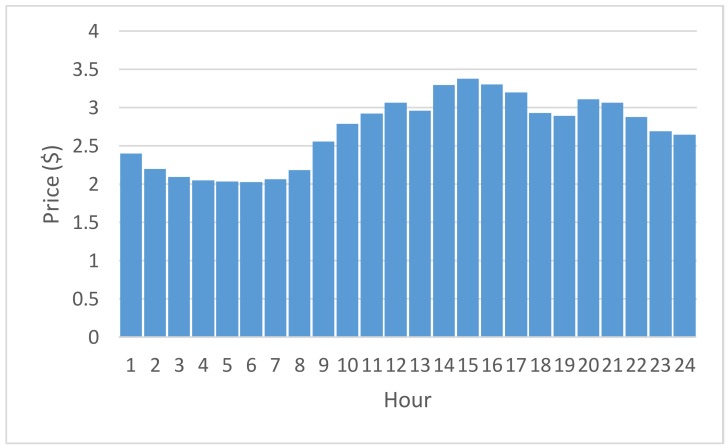
Assumed and simulated day-ahead RTP used to test the proposed method in this paper.

Experimentation is conducted in this section. The proposed constrained swarm intelligence (PSO)-based consumer-centric DSM method is examined in a household. No renewable energy generated can be included and used for the home appliances in the household. The home appliances used in the household are listed in Table 2. During the experimentation, data were gathered from the home appliances for 30 days; 1 day is divided into 1440 time slots (the time resolution is 1 min). During the load scheduling process in this paper, there is no need to manually pre-specify the physical characteristics of the enrolled schedulable home appliances in Table 3. That is, the energy disaggregation algorithm presented in [15] and applied on the gathered data is used to statistically and automatically identify the physical characteristics, *α_i_*, *β_i_*, and *l_i_*, of the enrolled schedulable home appliances by consumers with their past comfort satisfaction of using electricity for constrained PSO where randomly-generated particles encoded for *s_i_* and evaluated according to Equation (6) considering renewable energy resources under day-ahead predictable IBR-combined RTP are heuristically initialized in interval [*α_i_* − *δ_i_*, *β_i_* − *l_i_* + *δ_i_*]. The detailed optimization process by the constrained PSO is given later. Figure 2 shows the load profile of electric water boilers. The load profile of the schedulable home appliances is shifted according to DR pricing, during the optimization process. The energy disaggregation-piloting constrained PSO residential consumer DSM method proposed in this paper can be used to forecast short-term energy consumption based on the past trends of electricity usage by the resident(s) as well as minimize energy wastage of schedulable home appliances consuming electricity energy, as shown in Figure 2 as an example. The proposed method meta-heuristically optimizes the electricity cost while considering the user satisfaction under IP-based day-ahead IBR-combined RTP. The five-level IBR can be seen in Figure 3. The RTP assumed to be predicted ahead of the day, used to test the proposed method in this paper, and given in Figure 4 changes every hour. The accumulation time is scaled down from 1 month to 1 h. For instance, if the accumulative power consumption, 0.3322 kWh/h as an example, exceeds 0.1667 kWh/h, the received RTP within that hour is multiplied by a ratio of 1.276. The IBR imposes more expense and alleviates high PAR. *h* in Equation (6) becomes a dummy variable that total *H* in number equals 120 instead of 24 because 1 h is divided into 5 time slots. In the meantime, the IBR thresholds in every 12-min time slot can be computed and obtained. During the experimentation, the constrained PSO introduced in Section 3.2 is conducted for residential consumer-centric DSM. The parameters used by the constrained PSO are given in Table 3.

**Figure 5:**
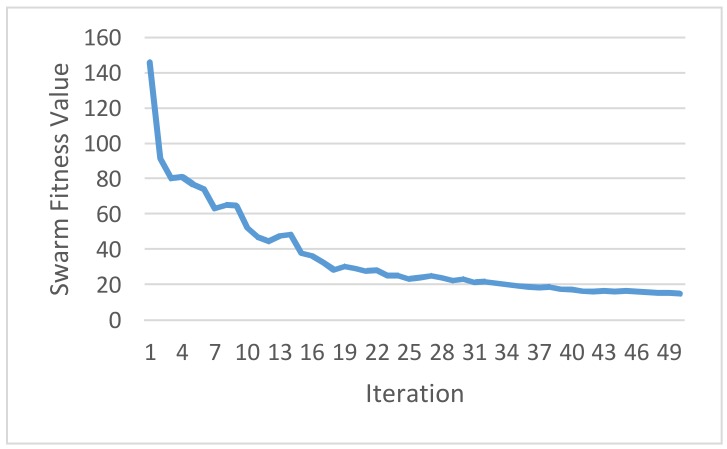
The optimal fitness value achieved and reported by the PSO was 15.070.

**Figure 6:**
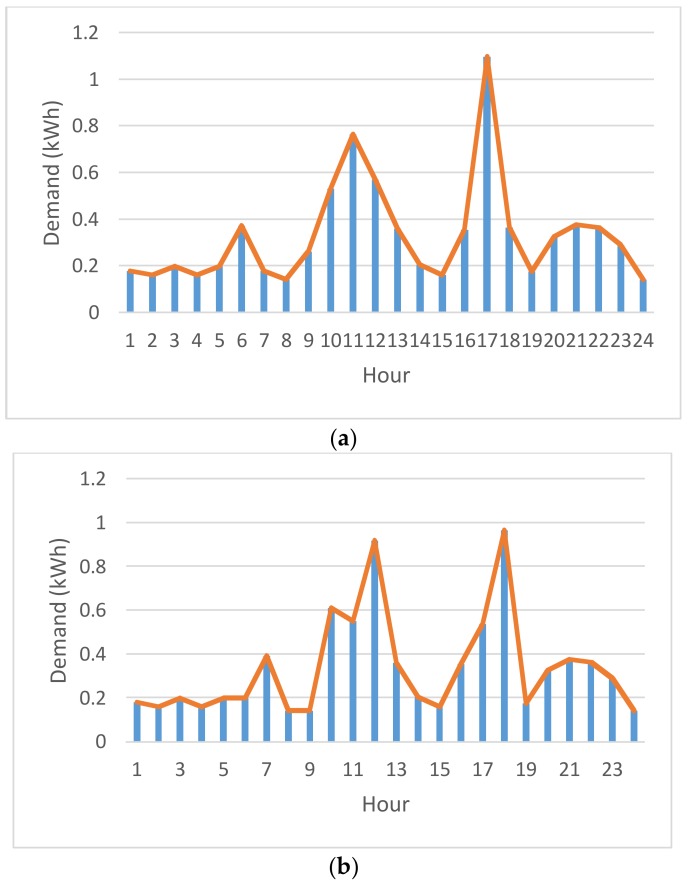
DSM/DR implementation with/without the proposed Residential consumer-centric DSM method: (**a**) the original load profile and (**b**) the resulting residential consumer-centric DSM solved by the PSO.

During the optimization process, each particle is encoded as a serial of 5 real numbers *s_i_* (refer to Table 3 in this case study), and is heuristically generated at random. The range of each weight coefficient is bounded by interval [−10, 10]. The PSO evaluates each particle in the current population based on the pre-clarified and problem-dependent objective function. The objective function clarified in this paper can be applied to domestic load scheduling with consideration of locally equipped renewable energy resources. The two objectives in Equation (6) were assigned an equal value of weights *w*_1_ and *w*_2_, respectively. In this paper, the PSO is implemented in R language, which is a free software environment for statistical computing and graphics and provides a free package repository to feature more than 11,800 available packages ranging from Machine Learning & Statistical Learning to Graphics for data science analytics and data visualization. Also, it is run on an ASUS ZENBOOK^TM^ Intel^®^ Core^TM^ i7 UX410UQ laptop computer. As shown in Figure 5, the optimal fitness value achieved and reported by the PSO was 15.070. Through the optimization process, s_i_* is [electric water boiler, steamer^a^, steamer^b^, steamer^c^, steamer^d^] = [1034.948, 361.1806, 589.5783, 671.9045, 1033.975]. According to the RTP given in Figure 4, the resulting residential consumer-centric DSM solved by the constrained PSO is shown in Figure 6. The whole-house load profile was re-shaped through the optimization process. The assumed and simulated predictable day-ahead RTP considered and used in this paper is based on an averaged daily load curve by Taipower (Taiwan Power Company, Taipei City, Taiwan), where the average daily load by houses in residential sectors of the power grid in Taiwan should be quite similar to that of the power generation cost by the utility (Taipower). Based on the past trends of load data automatically mined from the residents with their comfort satisfaction of using electricity, the home appliances scheduled for a new whole-house load profile (Figure 6b) and suggested to the residents with their past trends of using electricity are able to react to the assumed and simulated IP-based day-ahead IBR-combined RTP signal from the utility.

The economic benefit and phenomenal reduction of PAR achieved by the proposed method for residential consumer-centric DSM are summarized in Table 4.

As shown in Table 4, a phenomenal reduction in peak power consumption is achieved by 13.97%. The result shows the evidence of the proposed constrained PSO-based residential consumer-centric DSM method considering comfort satisfaction of using electricity to consumers based on the past trends of gathered load data in a smart home environment. Energy demands in residential dwellings are related to activity patterns of residents [29]. Appliances modeled in Equation (6) and managed by the residential consumer-centric DSM model in this paper can be further analyzed as activity patterns for human life-pattern identification.

## 5. Conclusions

In this paper, a residential consumer-centric DSM is first modelled. The model proposed in this paper will be featured with edge computing where a method of optimizing cloud computing technologies by driving computing capabilities at the edge/IoT data sources of the Internet emerges as one of the emerging trends in engineering technology. Then, in the model a new constrained swarm intelligence-based residential consumer-centric DSM method considering predictable day-ahead RTP with IBR and comfort satisfaction of using electricity to consumers based on past trends of load data in a smart home environment with the required minimal user intervention of setting the marginal parameter of each enrolled schedulable household appliance is implemented and validated, where electricity cost versus discomfort minimization along with peak demand reduction is realized. Nowadays, many households adopt the use of clean and sustainable renewable energy sources to satisfy their load demands. The objective function of Equation (6) clarified in this paper is considered alongside domestic load scheduling in the presence of locally equipped renewable energy resources contributing to grid safety and stability. A case study examining the proposed method has been demonstrated in this paper. The experimentation validates that the proposed method reflects electricity cost savings with consideration of user satisfaction of using electricity while achieving a phenomenal reduction, 13.97%, of PAR.

The work presented in this paper is comparative to the previous work in [15]; they will be examined and demonstrated further through multiple residential households in a downstream sector of a power grid leading a demonstration project of Regional Electrical Energy Integration, Dispatching and Ancillary Services in Taiwan in the future, where the energy disaggregation will be developed in a Big Data analytics way.

## Figures and Tables

**Table 1:** Nomenclature.

Abbreviation/Acronym	Expanded Form
ICT	Information and Communication Technologies
IoT	Internet of Things
DSM	Demand-Side Management
DR	Demand Response
RTP	Real-Time Pricing
IBR	Inclining Block Rates
PAR	Peak-to-Average Ratio
PSO	Particle Swarm Optimization
[*α_i_*, *β_i_*]	a time interval in which the *i*-th schedulable home appliance in a smart home environment was identified and expected statistically for use
*l_i_*	a time duration of the presence of the *i*-th schedulable home appliance
*s_i_*	the start instance of the *i*-th schedulable home appliance scheduled/optimized
*δ_i_*	a marginal parameter that the *i*-th schedulable home appliance is valid to be scheduled/optimized
*P^h^_renewable_*∙∆*_h_*	a term of locally generated renewable energy resources considered

**Table 2:** Monitored home appliances.

Home Appliance	Power Rating (kW)
electric rice cooker	1.10
electric water boiler	0.90
Steamer	0.80
TV	0.22
range hood	0.14
PC	0.35
hair dryer	1.20
washing machine	0.30
air conditioner	drawing variable power draws

**Table 3:** Statistically identified physical characteristics [15] of the enrolled schedulable home appliances.

Schedulable Home Appliances	[*α_i_*, *β_i_*]	[[${\tilde{l}}_{i}$]] ^1^	*δ_i_*
electric water boiler	[1035, 1071]	23	180
steamer ^a2^	[361, 379]	15	60
steamer ^b^	[589, 606]	15	90
steamer ^c^	[672, 721]	36	90
steamer ^d^	[1035, 1084]	24	90

^1^[[·]] rounds the averaged *l_i_* to the nearest integer. The averaged *l_i_* is less than *δ_i_*, and is obtained from the historical data statistically analyzed through the energy disaggregation. ^2^ steamer ^a–d^ represent the steamer is used four times in chronological order in one day.

**Table 4:** Economic benefit and phenomenal reduction of PAR achieved in this paper.

PSO-Based Residential Consumer-Centric DSM under IBR-Combined RTP	Unscheduled Demand	Scheduled Demand
Total Electricity Cost ($)	28.4482	28.2073 (−0.2409/improved by 0.85%)
PAR	3.3222	2.858 (−0.4642/improved by 13.97%)
